# Determination of the susceptibility of onychomycosis agents isolated in Sakarya to octenidine dihydrochloride and hypochlorous acid

**DOI:** 10.1007/s10096-026-05477-6

**Published:** 2026-04-17

**Authors:** Merve Gül, Ihsan Hakkı Çiftci, Gülay Erman, Ahmet Can Yağmur, Özlem Aydemir, İmdat Kılbaş, Mehmet Köroğlu

**Affiliations:** 1 Department of Medical Microbiology, Faculty of Medicine, Sakarya University, Sakarya, 54100 Turkey; 2 Health Services Education Research and Application Centre, Sakarya University, Sakarya, 54050 Turkey; 3 Department of Medical Biochemistry, Institute of Health Science, Sakarya University, Sakarya, 54050 Turkey; 4 Department of Dermatology, Darıca Farabi Training and Research Hospital, Ministry of Health, Kocaeli, Türkiye; 5 Dental Prosthesis Technology Program, Vocational School of Health Services, Fenerbahce University, Istanbul, Turkey

**Keywords:** Onychomycosis, Dermatophytes, Octenidine dihydrochloride, Hypochlorous acid, Terbinafine, Flow cytometry

## Abstract

The aim of this study was to investigate the susceptibility of onychomycosis agents to octenidine dihydrochloride (OCT-D) and hypochlorous acid (HOCl) antiseptics using agar dilution, tube dilution, and flow cytometry methods. In this study, onychomycosis agents including dermatophytes (*Trichophyton* spp., *Microsporum* spp., *Candida* spp.) and secondary agents such as *Aspergillus niger* and *Fusarium solani* were cultured on Sabouraud dextrose agar (SDA). Species identification was confirmed using MALDI-TOF MS. The minimum inhibitory concentration (MIC) values of OCT-D, HOCl, and terbinafine for dermatophytes grown on culture plates were determined using the agar dilution method and the broth microdilution (tube dilution) method in accordance with the European Committee on Antimicrobial Susceptibility Testing (EUCAST) guidelines. Following MIC determination, the minimum fungicidal concentration (MFC) was assessed. Based on the MFC results, flow cytometry analysis was performed using SYTO 9 and propidium iodide (PI) dyes to investigate the fungicidal effects of OCT-D, HOCl, and terbinafine.

As a result of antifungal susceptibility testing of onychomycosis agents, approximate MIC values obtained by the agar dilution method were 250 mg/L for OCT-D, 128 mg/L for HOCl, and 0.064 mg/L for terbinafine. Using the tube dilution method, approximate MIC values were 62.5 mg/L, 64 mg/L, and 0.032 mg/L for OCT-D, HOCl, and terbinafine, respectively. Flow cytometry-based viability analyses showed that the results obtained using the tube dilution method were more consistent. Since OCT-D and HOCl demonstrated MIC values far below their commercially available concentrations and exhibited high fungicidal activity, further studies suggest that these agents may be strong alternatives for topical treatment.

## Introduction

Onychomycosis, a fungal infection of the nail, is caused by a variety of organisms, including dermatophytes, yeasts, and non-dermatophyte molds [1, 2]. Dermatophytes, particularly *Trichophyton rubrum (T. rubrum)*, are recognized as the most common etiological agents of onychomycosis worldwide. In addition, yeasts such as *Candida* spp. and non-dermatophyte molds, including *Aspergillus fumigatus* and *Fusarium* spp., have also been reported as causative pathogens [3, 4]. Among non-dermatophyte molds, *Fusarium* species—especially *Fusarium solani (F.solani*), *Fusarium fujikuroi (F. fujikuroi)*, *Fusarium dimerum (F. dimerum)*, and *Fusarium oxysporum (F. oxysporum)*—have increasingly been identified in nail infections. Furthermore, mixed infections involving dermatophytes (e.g., *T. rubrum*) and non-dermatophyte molds have been documented, underscoring the importance of accurate identification of the causative agent for appropriate diagnosis and effective treatment. The prevalence of etiological agents may vary geographically, and non-*Candida albicans* species, particularly *Candida parapsilosis (C. parapsilosis)*, have been reported as common pathogens in certain regions [4, 5].

 [4, 5].

Current treatment methods for onychomycosis include a combination of pharmacological therapy and device-based techniques. Topical antifungals such as efinaconazole, tavaborole, and luliconazole are considered safer options, and novel agents such as ME-1111 and NP213 are currently under development [6]. Oral antifungals, including terbinafine, itraconazole, and griseofulvin, are recommended for moderate to severe cases, whereas topical treatments such as ciclopirox 8% nail lacquer and efinaconazole 10% solution are suitable for mild to moderate infections [7]. Photodynamic therapy (PDT) has emerged as a promising alternative, demonstrating high cure rates when used alone or in combination with antifungal agents [8]. Furthermore, advances in subungually administrable formulations have improved drug penetration through the nail plate, thereby enhancing treatment outcomes [9]. Overall, a tailored therapeutic approach that considers disease severity, patient-related factors, and treatment efficacy is essential for effective management of onychomycosis.

Non-pharmacological treatment options for onychomycosis include laser therapy and photodynamic therapy. Laser treatment using a 1,064-nm diode laser has demonstrated effectiveness, with clinical improvement rates ranging from 56% to 69% when used alone or in combination with topical antifungals [10]. In addition, PDT employing photosensitizers such as methylene blue or aminolevulinic acid (ALA), combined with appropriate pretreatment methods, offers a well-tolerated alternative for patients with contraindications to oral antifungal therapy or those who have not responded to conventional treatments [11]. Although alternative therapies such as tea tree oil, *Ageratina pichinchensis*, and Vicks VapoRub^®^ show potential benefits, large-scale clinical trials are required before their widespread endorsement [12]. Laser technologies, including Nd: YAG lasers, have demonstrated temporary clinical improvement and a reduction in positive fungal cultures, suggesting their potential role as antimycotic monotherapies [13].

Octenidine dihydrochloride (OCT-D) has been extensively studied for its antimicrobial properties [14]. It has demonstrated efficacy in inhibiting hyphal growth in opportunistic pathogenic fungi, indicating its potential as an antifungal therapeutic agent [15]. Additionally, octenidine has been shown to prevent plaque and gingivitis development in humans when used as the sole oral hygiene method for 21 days [16].

Terbinafine, an allylamine antifungal agent developed by Sandoz in 1984, is widely used worldwide for the systemic and topical treatment of dermatophyte infections, including tinea and onychomycosis. It inhibits squalene epoxidase, disrupting ergosterol synthesis, which suppresses fungal growth and induces cell death. Although point mutations in the SQLE gene can lead to terbinafine-resistant Trichophyton strains, terbinafine remains a well-established antifungal agent. In contrast, hypochlorous acid [MR6.1] is primarily used as a disinfectant [MR7.1]. When compared with terbinafine and hypochlorous acid, octenidine stands out due to its broad-spectrum antimicrobial activity, lack of systemic absorption through the skin, and significant inhibitory effects on hyphal growth and plaque formation [9, 13, 17].

## Materials and methods

Dermatophyte strains (*n* = 7) stored in the culture collection of T.C. Sakarya University Training and Research Hospital, as well as *Candida* species (*n* = 2) and *Fusarium* (1) and *Aspergillus (1)* species considered secondary pathogens of onychomycosis, were thawed and included in the study. Lactophenol cotton blue staining and matrix-assisted laser desorption/ionization time-of-flight mass spectrometry (MALDI-TOF MS) were used for species identification and confirmation.

The antifungal activities of OCT-D and HOCl were evaluated using agar dilution, tube dilution, minimum fungicidal concentration (MFC) determination, and viability analysis by flow cytometry. The prescription antifungal drug terbinafine was included as a reference compound to validate the experimental methodology. This study was approved by the T.C. Sakarya University Clinical Research Non-Interventional Ethics Committee on 18 February 2022 (Decision No: E-71522473-050.01.04-112733-37).

### Preparation of stock solutions

As no standardized EUCAST method exists for disinfectants, the following procedure was applied. Stock solutions (disinfectant solutions) were prepared according to formulas (1–2) [18]. For disinfectants, stock solutions were prepared at twice the commercially available concentrations, whereas terbinafine stock solutions were prepared at the concentrations specified in the EUCAST quality control (QC) Table (0.016 mg/L).

Hypochlorous acid (Microdacyn^®^, AquaBio, USA) is not commercially available in powder form; therefore, a commercially available HOCl solution was used directly as the stock solution. The antifungal effectiveness of HOCl was further evaluated using a time–kill assay.

1 $$\mathrm{Weight}\;(\mathrm g)=\lbrack\mathrm{volume}(\mathrm L)\times\mathrm{concentration}\;(\mathrm{mg}/\mathrm L)\rbrack/\mathrm{potency}\;\left(\mu\mathrm g/\mathrm g\right)$$

2 $$\mathrm{Volume}\;(\mathrm L)=\lbrack\mathrm{weight}(\mathrm g)\times\;\mathrm{potency}(\mathrm{mg}/\mathrm g)\rbrack/\;\mathrm{concentration}\;(\mathrm{mg}/\mathrm L)$$

### Preparation of inoculum

Fungal suspensions were prepared in sterile distilled water supplemented with 0.1% Tween 20 by collecting material from colonies grown in pure culture. Conidial suspensions were then obtained by filtration through sterile filter paper. The turbidity of the conidial suspensions was adjusted to 1.5–2.0 McFarland standards, whereas *Candida* spp. suspensions were adjusted to 0.5 McFarland standards. The prepared inocula were subsequently diluted 1:10. To ensure the viability and consistent concentration of conidia and yeast cells, all suspensions were inoculated within 30 min of preparation.

### Agar dilution

The agar dilution method was performed in duplicate, with incubation conducted both at room temperature and 37 °C in an incubator. Sabouraud dextrose agar (SDA) medium (250 mL) was prepared and sterilized. For OCT-D, the stock solution was prepared by dissolving the accurately weighed compound in sterile distilled water. Briefly, 300 mg of OCT-D was weighed using an analytical balance and dissolved in 50 mL of sterile distilled water to obtain the initial stock solution. Serial twofold dilutions were subsequently prepared from this stock solution for antifungal susceptibility testing. Twofold serial dilutions of the prepared stock solutions were then obtained using eight sterile containers. When the sterilized media cooled to 40–50 °C, the stock solutions were aseptically added through a syringe-mounted membrane filter with a pore size of 0.22 μm. To prevent bacterial contamination, gentamicin was added to each volumetric flask at a final concentration of 0.05 g/L. The media were then poured into Petri dishes to a uniform thickness of 4 mm, and the corresponding concentrations were labeled on each plate. For each strain, two disinfectant-free control media were included for sterility and growth control. All plates were inoculated using the prepared inoculum and a standardized inoculating loop. The inoculated Petri dishes were incubated at ambient temperature (25–28 °C) and in an incubator at 35–38 °C. Fungal growth was monitored for up to 5 days. If no growth was observed on the growth control plates, the experiment was repeated. The lowest concentration at which no visible growth was observed was defined as the minimum inhibitory concentration (MIC). In addition, a time–kill assay was performed to evaluate the effects of commercially available concentrations on strains with MIC values greater than 128 mg/L.

### Tube dilution

In this method, the EUCAST-recommended susceptibility testing procedure for dermatophytes was adapted to a tube dilution format and performed using RPMI 1640 liquid medium [18]. The medium was dispensed into previously prepared tubes at a volume of 5 mL per tube for each strain. Stock solutions, prepared at twice the final test concentration, were added to the first tube at a volume of 5 mL. Subsequently, 5 mL was transferred sequentially from one tube to the next and mixed thoroughly to obtain twofold serial dilutions. After mixing the final dilution, 5 mL was removed and discarded. No stock solution was added to the last two tubes, which served as growth control and sterility control, respectively. Following preparation of the inoculum, 50 µL was added to each test tube to achieve a final inoculum concentration of 10 µL/mL. The sterility control tube, containing only medium, was not inoculated. The prepared tubes were incubated at ambient temperature (25–28 °C) and in an incubator at 35–38 °C. Tubes were examined daily until visible growth was observed in the growth control tube. If no growth was detected in the growth control tube by the fifth day of incubation, the assay was repeated. The highest concentration at which no visible growth was observed was defined as the MIC. Samples from each tube were subsequently subcultured onto agar plates to assess contamination.

### Time–kill method

The effect of a commercially available concentration (0.02%) on HOCI dermatophytes was evaluated using the time–kill method [19]. Commercially available HOCl was dispensed into sterile tubes at a volume of 5 mL per tube. Freshly prepared inoculum (50 µL) was added to each tube and incubated at 37 °C.

Aliquots of 20 µL were collected from each tube at 0, 5, 10, 15, and 30 min and inoculated onto SDA. The absence of fungal growth after 5 min of exposure was considered indicative of sufficient antifungal activity. If no growth was observed on the growth control plates by the fifth day of incubation, the assay was repeated.

### Minimum fungicidal concentration

In the tube dilution method, 10 µL aliquots were collected from the tubes corresponding to the MIC values and from other tubes in which no visible growth was observed. The aliquots were inoculated onto two antifungal-free SDA plates and evenly spread using a sterile loop. This procedure was repeated for all 11 strains. For evaluation, the inoculated plates were incubated for five days at both ambient temperature and in an incubator. Growth was assessed daily. If no growth was observed on the growth control plate by the fifth day of incubation, the assay was repeated. The MFC was defined as the lowest concentration at which no fungal growth was observed.

### Flow cytometry method

Flow cytometric analysis was performed using a FACSCalibur flow cytometer (Becton Dickinson, USA). Test tubes were prepared at commercially available concentrations of OCT-D (1000 mg/L) and HOCl (200 mg/L), as well as at concentrations corresponding to species-specific MFC values determined in previous terbinafine studies. In parallel, growth and sterility control tubes were prepared for each antifungal agent. *Candida* inoculum were adjusted to a turbidity of 1.0–1.5 McFarland to ensure sufficient fungal cell density and consistent growth conditions for accurate determination of antifungal susceptibility. The inoculum was added to each tube at a volume of 10 µL per milliliter of medium and cultured accordingly. Following inoculation, 1 mL of suspension was collected at 0 h and transferred to flow cytometry reading tubes. The remaining suspension was incubated for 5 and 24 h. To remove residual disinfectants, the 1 mL suspensions were washed twice with phosphate-buffered saline (PBS) by centrifugation at 4000 rpm for 20 min. After washing, 2 µL of SYTO 9 and 2 µL of propidium iodide (PI) were added to each tube. The samples were incubated in the dark for 15–30 min. At the end of the incubation period, flow cytometric analysis was performed. This procedure was repeated for samples collected at 5 and 24 h. Yeast cell suspensions were analyzed using the SSC-A/FITC-A channels, and data were processed using BD FACSDiva software. SYTO 9 staining was used to determine the total cell population, whereas PI staining was used to identify non-viable (dead) cells.

## Results

Microscopic identification was performed using the reference source *Medically Important Fungi: A Guide to Identification* [20]. Definitive species identification was subsequently confirmed by MALDI-TOF MS. Several *Fusarium* spp. isolates that had been misidentified by microscopic examination were correctly identified using MALDI-TOF MS, underscoring the importance of verification with automated identification systems. The results are summarized in Table 1.

**Table 1:** Comparison of methods used in identification of strains

Isolat No	Microscopic identification with Lactophenol Cotton Blue		Identification with MALDI-TOF MS
1*		*Trichphyton* spp.	Identification could not be made
2*		*Trichphyton* spp.	*T. erinaceii*
3*		*Microsporum* spp.	*M. gypseum*
4*		*Microsporum* spp.	Identification could not be made
5		*Trichphyton* spp.	*T. rubrum*
6		*Microsporum canis*	*M. canis*
7		*Trichphyton* spp.	*T. violaceum*
8		*Trichphyton* spp.	*Fusarium solani complex*
9		*Aspergillus niger*	*Aspergillus niger*
10		*Candida albicans*	*C. albicans*
11		*Candida* spp.	*C. parapsilosis*

*These are standard strains found in the National Pathogenic Fungi Collection (NCPF)

Agar dilution tests demonstrated that OCT-D exerted a lethal effect even at concentrations well below the commercially available concentration. In the agar dilution assay performed using serial dilutions of the commercial HOCl concentration (200 ppm), primary pathogens were eliminated at lower concentrations, whereas secondary agents (*Aspergillus* spp., *Fusarium* spp., and *Candida* spp.) were able to grow at these same concentrations. In the terbinafine assay, contrary to the value reported in the guideline, the MIC was greater than 0.016 mg/L for all strains except strains 2 and 8. Stock solutions of the antifungal agents were prepared and subsequently subjected to stepwise serial dilution. MIC₅₀ was defined as the concentration inhibiting 50% of the isolates, whereas MIC₉₀ was defined as the concentration inhibiting 90% of the isolates. The results of these analyses are presented in Table 2.

**Table 2:** MIC range and MIC values investigated by agar dilution for OCT-D, HOCl disinfectants and Terbinafine antifungals

SPECIES	OCT-D MIC	HOCl MIC	Terbinafine MIC (mg/l)^b^
	(mg/l)	(mg/l)	
*1. T. tonsurans*	< 31.25	16	0.032
*2. T. erinaceii*	< 31.25	32	0.016
*3. M. gypseum*	125	64	0.032
*4. M. nanum* ^*a*^	< 31.25	8	0.064
*5. T. rubrum*	< 31.25	32	0.032
*6. M. canis*	< 31.25	64	0.032
*7. T. violaceum*	< 31.25	32	0.016
*8. Fusarium solani* complex	500	> 128	> 32
*9. Aspergillus niger*	250	128	16
*10. C. albicans*	< 31.25	> 128	> 32
*11. C. parapsilosis*	125	> 128	32

“-“ No growth was observed. Lower than the tested value

^a^Since M. nanum grows late in liquid medium, it was expected to grow until the 5th day

^b^The MIC range of the strains whose MIC values are stated in the literature were adjusted and tested according to the specified MIC range

In tube dilution assays, the MIC of OCT-D for both dermatophytes and secondary agents of onychomycosis was found to be below 31.25 mg/L, which is well below the commercially available concentration (0.1%). For HOCl, three primary dermatophyte isolates exhibited MIC values of 128 mg/L, while four isolates showed MIC values of 64 mg/L. In contrast, the MIC values of HOCl for non-dermatophyte agents were greater than 128 mg/L. MIC evaluation for terbinafine revealed that, among primary dermatophyte agents, one isolate exhibited an MIC of 0.064 mg/L, three isolates had MIC values of 0.032 mg/L, and three isolates showed MIC values of 0.016 mg/L. Among non-dermatophyte agents, two isolates exhibited MIC values greater than 32 mg/L, whereas the remaining isolates showed MIC values of 16 mg/L and 8 mg/L. The results obtained using this method are presented in Table 3.

**Table 3:** Tube dilution test MIC value ranges, found MIC values and MFC values for OCT-D, HOCl disinfectants and Terbinafine antifungal

SPECIES		OCT-D (mg/l)	HOCl (mg/l or minute)	Terbinafine (mg/l)^b^
*1. T. tonsurans*	MIC	< 31.25	128	0.032
	MFC	NG	128	0.032
*2. T. erinaceii*	MIC	< 31.25	64	0.016
	MFC	NG	64	0.016
*3. M. gypseum*	MIC	< 31.25	64	0.032
	MFC	NG	64	0.032
*4. M. nanum* ^*a*^	MIC	< 31.25	64	0.064
	MIC	NG	64	0.064
*5. T. rubrum*	MIC	< 31.25	128	0.032
	MIC	NG	> 128^c^	0.032
*6. M. canis*	MIC	< 31.25	128	0.016
	MFC	NG	> 128^c^	0.016
*7. T. violaceum*	MIC	< 31.25	64	0.016
	MFC	NG	64	0.016
*8. Fusarium solani* complex	MIC	< 31.25	> 128	> 32
	MFC	62.5	5 min	> 32
*9. Aspergillus niger*	MIC	< 31.25	> 128	8
	MFC	-	10 min	16
*10. C. albicans*	MIC	< 31.25	> 128	> 32
	MFC	NG	5 min	> 32
*11. C. parapsilosis*	MIC	< 31.25	> 128	16
	MFC	NG	5 min	16

“-“ No growth was observed. Lower than the tested value

^a^Since *M. nanum* grows late in liquid medium, it was expected to grow until the 5th day

^b^The MIC range of the strains whose MIC values are stated in the literature were adjusted and tested according to the specified MIC range

^c^In the time–kill test, complete eradication of the strains was observed after 10 min of contact with the disinfectant

MFC evaluation was conducted based on the previously determined MIC values. In the MFC assay for OCT-D, no fungal growth was observed at any of the concentrations tested. In MFC assays performed for HOCl, the MFC values were identical to the MIC values for most strains; however, for the fifth and sixth strains, the MFC values were higher than the corresponding MIC values. In the time–kill evaluation conducted to assess the effects of commercially available concentrations, no fungal growth was observed after 10 min of exposure. For terbinafine, the MFC values were identical to the MIC values. The results obtained using this method are presented in Table 3.

**Fig. 1:**
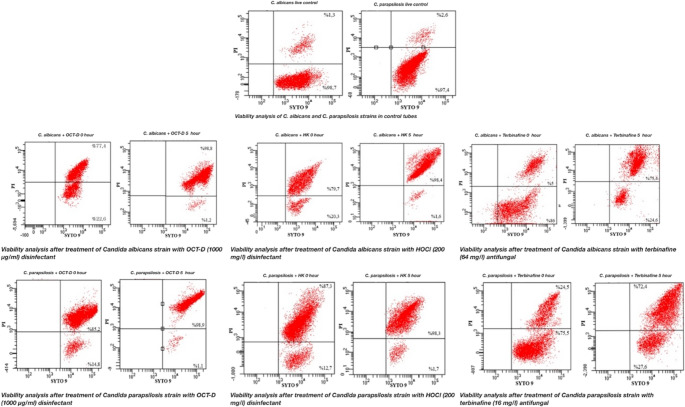
Graphs obtained in flow cytometry analyses for *C. albicans* and *C. parapsilosis* species

*Candida albicans* and *Candida parapsilosis* strains were evaluated for viability at 0 and 5 h following treatment with commercially available concentrations of disinfectants and terbinafine. The tested agents demonstrated high efficacy from the initial contact with *Candida* species. The results of this analysis are presented in Table 4; Fig. 1.

**Table 4:** Live and dead yeast ratios obtained from analysis by flow cytometry

Disinfectant & Antifungal to Test	Specıes	Vitality Control		0. hour		24. hour	
		% Live	% Dead	% Live	% Dead	% Live	% Dead
OCT-D	*Candida* *albicans*	98.7	1.3	22.6	77.4	1.2	98.8
HOCl				20.3	79.7	1.6	98.4
Terbinafine				86	5	24.6	75.4
OCT-D	*Candida* *parapsilosis*	97.4	2.6	14.8	85.2	1.1	98.9
HOCl				12.7	87.3	1.7	98.3
Terbinafine				75.5	24.5	27.6	72.4

## Discussion

Onychomycosis is a fungal infection of the nail caused by dermatophytes or non-dermatophyte molds or yeasts. These infections caused by dermatophytes have been shown to be difficult to treat and respond poorly to oral medications [12]. Nowadays, due to factors such as prolonged treatment duration, poor patient adherence, and potential drug–drug interactions, alternative antifungal treatment strategies that are faster, low-cost, and short-term are increasingly being explored [21, 22]. In the present study, disinfectants with demonstrated antifungal efficacy were selected because they represent potential candidate agents under these circumstances. Fungal identification remains challenging due to the need for experienced personnel capable of making accurate morphological distinctions using traditional microscopic methods and preparing specimens appropriately [23]. More reliable identification can be achieved by verifying potential misidentifications obtained through microscopic examination using automated systems such as MALDI-TOF MS. Therefore, further studies are required to enhance the reliability of automated identification systems and to ensure accurate and reproducible results.

In the literature and EUCAST guidelines, no sufficient data or clearly defined values were found regarding agar dilution assays investigating the effectiveness of OCT-D and HOCl against dermatophytes. Moreover, the agar dilution method is highly time-consuming. We believe that the findings of the present study may provide valuable guidance for future research in this area. The literature review indicated that the clinical concentration of OCT-D ranges between 0.05% and 0.1% (w/w), corresponding to 0.1 g of octenidine dihydrochloride per 100 g of the commercially available formulation [24, 25]. It was also reported that the commercial concentration of HOCl is equivalent to 200 ppm [26, 27]. Our results demonstrated that OCT-D was effective against dermatophyte agents even at a concentration as low as 0.000625%. In the agar dilution assay, HOCl showed antifungal activity comparable to that of OCT-D against the primary agents of onychomycosis; however, it did not exhibit sufficient efficacy against secondary agents.

In a study conducted on sixty dermatophyte isolates using azole group drugs, the mean MIC value of terbinafine was reported as 0.2 mg/L for all isolates using the agar dilution method, whereas this value was 0.01 mg/L when determined by the microdilution method. The authors stated that MIC values obtained by the microdilution test are more appropriate for determining the drug dosage to be administered to patients [28]. Consistent with these findings, the results of the present study demonstrated that secondary agents of onychomycosis, such as *Fusarium* spp., exhibited resistance to terbinafine even in agar dilution assays. Therefore, it is suggested that antifungal resistance studies focusing on secondary agents of onychomycosis should be expanded. Furthermore, a review study recommended that microdilution tests should be used more effectively than agar dilution tests, as they provide more accurate results when determining the appropriate drug dosage for patients [29].

In a study conducted with OCT-D and amphotericin B, the strong antifungal effect of OCT-D against *Candida* species was demonstrated [30]. In an in vitro study evaluating disinfectants at an 80% concentration, povidone–iodine and octenidine dihydrochloride exhibited high fungicidal activity against clinical isolates of *T. rubrum*, *T. interdigitale*, *C. parapsilosis*, and *C. albicans*. However, the authors emphasized that fungicidal activity against *Aspergillus* spp. was weaker [17]. In another study investigating the antifungal activity of OCT-D, it was reported that OCT-D was effective against onychomycosis agents such as *Candida* spp. even at concentrations (0.0004–0.00005%) well below the clinically recommended range (0.05–0.1%) [31]. Consistent with these findings, the results of the tube dilution assay performed in the present study demonstrated that the MIC values of OCT-D were markedly lower than the commercially available concentration. These low MIC values suggest that OCT-D may represent a promising alternative for topical antifungal therapy. However, an important question remains regarding whether this disinfectant can effectively penetrate from the surface of dermatophyte-infected nails to the subungual region at therapeutically appropriate concentrations.

A study was conducted to investigate the effectiveness of HOCl using the time–kill method against *Trichophyton verrucosum* and *Epidermophyton floccosum*. According to this study, the growth of *E. floccosum* decreased with increasing exposure time, and no growth was observed at the 1/64 dilution after 8 h of treatment. For *T. verrucosum*, no growth was reported at any dilution after 15 min of exposure [32]. In another study, microdilution assays performed for species such as *Fusarium* spp., *Aspergillus* spp., and *Candida* spp. resulted in a 90% kill rate after 1 min of exposure at a concentration of 0.01% (w/w) [33]. In the present study, MIC values for dermatophytes ranged between 64 and 128 mg/L. Organisms with MIC values exceeding the HOCl concentration of 0.01% (128 mg/L) were identified as fungal species (*Fusarium* spp., *Aspergillus* spp., and *Candida* spp.) that act as secondary agents of onychomycosis. The antifungal activity of strains with MIC values greater than 128 mg/L was further evaluated using the time–kill method at commercially available concentrations, and no fungal growth was observed in any strain after 10 min of exposure to HOCl.

In the microdilution assay for terbinafine, the EUCAST-recommended quality control value of 0.016 mg/L for *T. rubrum* was tested. In a study conducted in 2018, the MIC of terbinafine against *Trichophyton* spp., the primary agents of onychomycosis, was reported to be 0.06 µg/mL. Among other antifungal agents tested, terbinafine was reported to exhibit the lowest MIC values [34]. According to data obtained in another study conducted in 2018, terbinafine and itraconazole were identified as the antifungal agents with the highest activity against *Microsporum canis*, regardless of the susceptibility testing method used [35]. A study conducted in China in 2021 reported that the susceptibility profiles of *Trichophyton rubrum*, one of the most common dermatophytes, tend to vary regionally [36]. In addition, terbinafine, which was used as a positive control in a previous study, has been shown to play an active role in inducing apoptosis in *T. rubrum* [37]. Consistent with reports from many studies involving dermatophytes, antifungal resistance profiles in the present study also differed and were found to be approximately twofold higher than the values reported by EUCAST. Although the use of the tube dilution method for antifungal susceptibility testing should be taken into account, the observed differences in resistance profiles may be attributed to the development of antifungal resistance in the tested strains.

In a study evaluating the antiseptic efficacy of triclosan, povidone–iodine, octenidine dihydrochloride, polyhexanide, and chlorhexidine digluconate, it was reported that the MIC value (0.001 mg/mL) and the MFC value of OCT-D were identical for *Candida* species [26]. In another study in which polyhexanide, OCT-D, and NaClO/HOCl antiseptics were tested, the MFC (% v/v) values for *Candida albicans* were reported as 0.36% (± 0.18) for polyhexanide, 0.09% for OCT-D, and > 25% for NaClO/HOCl [38]. In a microdilution study evaluating antifungal susceptibility of *Trichophyton* spp., *Microsporum canis*, and *Candida* spp. using terbinafine and itraconazole, most dermatophyte isolates were found to be highly susceptible, whereas the MFC values for *Candida* species ranged between 1 and 2 mg/L [39]. In another study conducted with *Trichophyton rubrum* and *Trichophyton mentagrophytes* strains, the MIC (0.31 mg/L) and MFC (0.31 mg/L) values of essential oils and terbinafine were reported to be identical [40]. Consistent with the literature, the MIC and MFC values obtained in the present study were closely aligned. OCT-D and HOCl demonstrated fungicidal activity against the tested dermatophytes, and the MFC values were found to be equal to or slightly higher than the corresponding MIC values, in agreement with previously reported findings.

In one study, the lethal effects of metalworking fluids on *Fusarium* spp. were investigated using the LIVE/DEAD BacLight kit. While nucleic acids of both viable and non-viable fungal cells were stained with SYTO 9, it was reported that only the nucleic acids of dead fungal cells were stained with propidium iodide (PI). Although a small proportion of dead fungi was observed at the initial time point, complete fungal death was detected after 24 h of exposure [41]. In another study investigating apoptosis in *T. rubrum* using flow cytometric analysis, terbinafine was shown to exert strong antifungal activity and to induce apoptosis. In that study, Annexin V, which binds to phosphatidylserine exposed on fragmented cell membranes, and PI, which binds selectively to the nucleic acids of non-viable cells, were used [37]. Consistent with these findings, the results of the flow cytometric analysis performed in the present study for *C. albicans* and *C. parapsilosis* are presented in Table 4 and Supplementary Fig. 1. The pronounced effects of disinfectants observed even at the initial time point support the MIC findings. Moreover, the results of flow cytometry and viability analyses suggest that this approach can be applied not only to bacteria or single-cell organisms but also to fungi and yeasts with hyphal structures. Nevertheless, further data are required to optimize flow cytometric protocols for fungi containing hyphae.

## Conclusions

Research and analysis of antifungal resistance in fungi remain neglected in many laboratories due to the time-consuming and labor-intensive nature of these procedures. However, it should be emphasized that all fungi causing infections have the potential to develop resistance to antifungal agents, similar to bacteria. Therefore, standardized methods for fungal identification and antifungal susceptibility testing are essential. Effective fungal identification is likely to be achieved through method standardization, expansion of microorganism reference libraries, and the development of novel and rapid procedures for hyphae-forming fungi, particularly within automated systems such as MALDI-TOF MS. In addition, the differences observed between agar dilution and microdilution results in both the literature and the present study highlight the need for further research to establish standardized and reliable susceptibility testing methods.

As a result of the present study, OCT-D and HOCl were shown to be at least as effective as currently used antifungal agents against onychomycosis pathogens. Moreover, both agents exhibited fungicidal activity even at concentrations far below those that are commercially available. In light of these findings, OCT-D and HOCl may represent promising alternatives for the topical treatment of onychomycosis.

However, for OCT-D and HOCl to effectively reach onychomycosis agents located in the deeper layers of the nail, their combined application with penetration-enhancing agents may improve treatment efficacy. Therefore, further studies are required to investigate the combined use of antifungal agents with penetration enhancers while preserving their chemical stability and integrity.

At the same time, to enable safe and practical use in humans, the cytotoxic effects of combinations of OCT-D and HOCl formulated with penetration-enhancing agents should be thoroughly investigated. Therefore, based on the findings of the present study, further extensive and comprehensive studies are warranted.

## Data Availability

The raw data supporting the conclusions of this article will be made available by the authors on request.

## References

[1] Gupta PK, Ranjan S (2023) A case study of onychomycosis caused by *Aspergillus fumigatus*. Galore Int J Health Sci Res 7:1–4. 10.52403/gijhsr.20220701

[2] Gupta AK, Taborda VBA, Taborda PRO, Shemer A, Summerbell RC, Nakrieko KA (2020) High prevalence of mixed infections in global onychomycosis. PLoS ONE 15:e0239648. 10.1371/journal.pone.0239648 32991597 10.1371/journal.pone.0239648 PMC7523972

[3] Genç GE, Nuriyev K, Küçükkaya S, Şatana D, Uzun M, Erturan Z (2022) Causative agents of onychomycosis and associated factors: a 15-year study. Med Mycol 60:P479. 10.1093/mmy/myac072.p479

[4] Veiga FF, de Castro-Hoshino LV, Rezende PST, Baesso ML, Svidzinski TIE (2022) Insights on the etiopathogenesis of onychomycosis by dermatophyte, yeast and non-dermatophyte mould in ex vivo model. Exp Dermatol 31:1810–1814. 10.1111/exd.14643 35818750 10.1111/exd.14643

[5] Tyagi S, Kaur N, Rawat R D (2021) A study of etiology and epidemiology of onychomycosis from a tertiary care hospital in North India. Int J Res Med Sci 9:559–564. 10.18203/2320-6012.ijrms20210442

[6] Gregoriou S, Kyriazopoulou M, Tsiogka A, Rigopoulos D (2022) Novel and investigational treatments for onychomycosis. J Fungi 8:1079. 10.3390/jof8101079 10.3390/jof8101079 PMC9604567 36294644

[7] Falotico JM, Lipner SR (2022) Updated perspectives on the diagnosis and management of onychomycosis. Clin Cosmet Invest Dermatology 15:1933. 10.2147/CCID.S362635 10.2147/CCID.S362635 PMC9484770 36133401

[8] Navarro-Bielsa A, Gracia-Cazaña T, Robres P, Lopez C, Calvo-Priego MD, Aspiroz C, Gilaberte Y (2022) Combination of photodynamic therapy and oral antifungals for the treatment of onychomycosis. Pharmaceuticals (Basel) 15:722. 10.3390/ph15060722 35745641 10.3390/ph15060722 PMC9227606

[9] Aggarwal R, Targhotra M, Kumar B, Sahoo PK, Chauhan MK (2020) Treatment and management strategies of onychomycosis. J Mycol Med 30:100949. 10.1016/j.mycmed.2020.100949 32234349 10.1016/j.mycmed.2020.100949

[10] Weber GC, Firouzi P, Baran AM, Bölke E, Schrumpf H, Buhren BA, Homey B, Gerber PA (2018) Treatment of onychomycosis using a 1064-nm diode laser with or without topical antifungal therapy: a single-center, retrospective analysis in 56 patients. Eur J Med Res 23:1–8. 10.1186/s40001-018-0340-y 30355363 10.1186/s40001-018-0340-y PMC6199788

[11] Nickles MA, Lio PA, Mervak JE (2022) Complementary and alternative therapies for onychomycosis: a systematic review of the clinical evidence. Skin Appendage Disorders 8:269–279. 10.1159/000521703 35983465 10.1159/000521703 PMC9274952

[12] Lipner SR, Vlahovic T, Ghannoum MA, Elewski B, Joseph WS (2024) Dermatophytomas in onychomycosis: a scoping review of prevalence, diagnosis, and treatment. J Am Podiatr Med Assoc 114:22–161. 10.7547/22-161 38753536 10.7547/22-161

[13] Gupta AK, Wang T, Cooper EA, Summerbell RC, Piguet V, Tosti A, Piraccini BM (2024) A comprehensive review of nondermatophyte mould onychomycosis: Epidemiology, diagnosis and management. J Eur Acad Dermatol Venereol 38:480–495. 10.1111/jdv.19644 38010049 10.1111/jdv.19644

[14] Hübner NO, Siebert J, Kramer A (2010) Octenidine dihydrochloride, a modern antiseptic for skin, mucous membranes and wounds. Skin Pharmacol Physiol 23:244–258. 10.1159/000314699 20484966 10.1159/000314699

[15] Köck R, Denkel L, Feßler AT, Eicker R, Mellmann A, Schwarz S, Geffers C, Hübner NO, Leistner R (2023) Clinical evidence for the use of octenidine dihydrochloride to prevent healthcare-associated infections and decrease *Staphylococcus aureus* carriage or transmission: a review. Pathogens 12:612. 10.3390/pathogens12040612 37111498 10.3390/pathogens12040612 PMC10145019

[16] Seegräber M, Ruzicka T, Wollenberg A (2017) Persistierende Rötung und Induration nach Wundspülung mit Octenidin. Der Hautarzt 68:326–328. 10.1007/s00105-016-3895-y 10.1007/s00105-016-3895-y 27826662

[17] Fang T, Ji Z, Lu H, Jiang Y (2022) Identification of octenidine (dihydrochloride) inhibiting fungal filamentation by the repurposing approach. Med Mycol 60:P062. 10.1093/mmy/myac072.p062

[18] Arendrup MC, Kahlmeter G, Guinea J, Meletiadis J (2021) How to perform antifungal susceptibility testing of microconidia-forming dermatophytes following the EUCAST reference method E.Def 11.0, exemplified by *Trichophyton*. Clin Microbiol Infect 27:55–60. 10.1016/j.cmi.2020.08.042 32916260 10.1016/j.cmi.2020.08.042

[19] Kılbaş İ, Hatipoğlu H, Kılıç U, Kahraman Kılbaş EP, Koroğlu M, Altındiş M (2021) Investigation of the synergic effect of the colistin/sulbactam combination in carbapenem-resistant *Acinetobacter baumannii* complex strains with time-kill and checkerboard methods. FLORA - J Infect Clin Microbiol 26:151–162. 10.5578/flora.20219916

[20] Larone DH, Walsh TJ, Hayden RT (2018) Larone’s Medically Important Fungi: A Guide to Identification. 6th ed. ASM Press, Washington, DC. ISBN: 9781555819839

[21] Gül M, Çiftci İ (2022) Onikomikoz ve tedavi yaklaşımları. Sağlık Akademisi Kastamonu 7:587–612. 10.25279/sak.1053918

[22] Singal A, Khanna D (2011) Onychomycosis: diagnosis and management. Indian J Dermatol Venereol Leprol 77:659–670. 10.4103/0378-6323.86475 22016272 10.4103/0378-6323.86475

[23] Shamly V, Kali A, Srirangaraj S, Umadevi S (2014) Comparison of microscopic morphology of fungi using lactophenol cotton blue, iodine glycerol and Congo red formaldehyde staining. J Clin Diagn Res 8. 10.7860/JCDR/2014/8521.4535 10.7860/JCDR/2014/8521.4535 PMC4149071 25177565

[24] Amin N, Shenoy MM, Pai V (2023) Clinical and mycological characterization of chronic and recurrent dermatophytes using various staining and microscopic methods. J Pure Appl Microbiol 17:2598–2608. 10.22207/JPAM.17.4.59

[25] Octenisept^®^ (2025) Schülke. Available online: https://www.schuelke.com/intl-en/products/octenisept.php. Accessed 5 Jan 2025

[26] Oktenidin dihidroklorür (2023) Ataman Kimya A.Ş. Available online: https://www.ataman-chemicals.com/urunler/oktenidin-dihidroklorur-519.html. Accessed 4 Dec 2023

[27] Koburger T, Hubner NO, Braun M, Siebert J, Kramer A (2010) Standardized comparison of antiseptic efficacy of triclosan, PVP-iodine, octenidine dihydrochloride, polyhexanide and chlorhexidine digluconate. J Antimicrob Chemother 65:1712–1719. 10.1093/jac/dkq212 20551215 10.1093/jac/dkq212

[28] Crystalin (2025) İnsan sağlığı kimyasal ve farmakolojik özellikleri. Available online: https://nhp.com.tr/sayfa/crystalin-insan-sagligi-kimyasal-ve-farmakolojik-ozellikleri. Accessed 5 Jan 2025

[29] Mota CRA, Miranda KC, Lemos JDA, Costa CR, Hasimoto e Souza LK, Passos XS, Menesese Silva H, Silva MDRR (2009) Comparison of in vitro activity of five antifungal agents against dermatophytes using agar dilution and broth microdilution methods. Rev Soc Bras Med Trop 42:250–254. 10.1590/S0037-86822009000300003 19684970 10.1590/s0037-86822009000300003

[30] Sanchez Armengol E, Harmanci M, Laffleur F (2021) Current strategies to determine antifungal and antimicrobial activity of natural compounds. Microbiol Res 252:126867. 10.1016/j.micres.2021.126867 34521051 10.1016/j.micres.2021.126867

[31] Sakarya S, Günay N, Öztürk ŞB, Ertuğrul B (2013) Dermatofitozların tedavisinde yeni bir güçlü ajan: Hipokloröz asit (Crystalin^®^). XVI KLİMİK KONGRESİ. http://www.nhp.com.tr/referanslar/referans_14.pdf. Erişim tarihi: 21 Mayıs 2023

[32] Silva-Neves V, Hugo V, Alves P, Amado JC, Pais-Vieira C, Sousa F, Cerqueira F, Pinto E, Pais-Vieira M (2021) Quality of life and therapeutic regimen management in onychomycosis patients and in vitro study of antiseptic solutions. Sci Rep 11:1–10. 10.1038/s41598-021-92111-4 34140577 10.1038/s41598-021-92111-4 PMC8211768

[33] Ponnachan P, Vinod V, Pullanhi U, Varma P, Singh S, Biswas R, Kumar A (2019) Antifungal activity of octenidine dihydrochloride and ultraviolet-C light against multidrug-resistant *Candida auris*. J Hosp Infect 102:120–124. 10.1016/j.jhin.2018.09.008 30261239 10.1016/j.jhin.2018.09.008

[34] Odorcic S, Haas W, Gilmore MS, Dohlman CH (2015) Fungal infections after Boston type 1 keratoprosthesis implantation. Cornea 34:1599–1605. 10.1097/ICO.0000000000000639 26488624 10.1097/ICO.0000000000000639 PMC4636962

[35] Kulkarni SS, Bhakre JB, Damle AS (2018) In vitro susceptibility testing of four antifungal drugs against fungal isolates in onychomycosis. Int J Res Med Sci 6:2774–2780. 10.18203/2320-6012.ijrms20183268

[36] Aneke CI, Otranto D, Cafarchia C (2018) Therapy and antifungal susceptibility profile of *Microsporum canis*. J Fungi 4:107. 10.3390/jof4030107 10.3390/jof4030107 PMC6162526 30189676

[37] Jiang Y, Luo W, Verweij PE, Song Y, Zhang B, Shang Z, Al-Hatmi AMS, Ahmed SA, Wan Z, Li R (2021) Regional differences in antifungal susceptibility of the prevalent dermatophyte *Trichophyton rubrum*. Mycopathologia 186:53–70. 10.1007/s11046-020-00515-z 33313977 10.1007/s11046-020-00515-z PMC7946697

[38] Li ZJ, Abula A, Abulizi A, Wang C, Dou Q, Maimaiti Y, Abudouaini A, Huo SX, Aibai S (2020) Ellagic acid inhibits *Trichophyton rubrum* growth via affecting ergosterol biosynthesis and apoptotic induction. Evidence-Based Complement Altern Med 2020:7305818. 10.1155/2020/7305818 10.1155/2020/7305818 PMC7641703 33193798

[39] Krasowski G, Junka A, Paleczny J, Czajkowska J, Makomaska-Szaroszyk E, Chodaczek G, Majkowski M, Migdał P, Fijałkowski K, Kowalska-Krochmal B (2021) In vitro evaluation of polihexanide, octenidine and NaClO/HClO-based antiseptics against biofilm formed by wound pathogens. Membr (Basel) 11:62. 10.3390/membranes11010062 10.3390/membranes11010062 PMC7830887 33477349

[40] Hazen KC (1998) Fungicidal versus fungistatic activity of terbinafine and itraconazole: an in vitro comparison. J Am Acad Dermatol 38:S37–S41. 10.1016/S0190-9622(98)70482-7 9594935 10.1016/s0190-9622(98)70482-7

[41] Trifan A, Luca SV, Bostănaru AC, Brebu M, Jităreanu A, Cristina RT, Skalicka-Woźniak K, Granica S, Czerwińska ME, Kruk A (2021) Apiaceae essential oils: boosters of terbinafine activity against dermatophytes and potent anti-inflammatory effectors. Plants 10:2378. 10.3390/plants10112378 34834740 10.3390/plants10112378 PMC8623916

